# Parenteral nutrition improves nutritional status, autonomic symptoms and QoL in patients with TTR-FAP

**DOI:** 10.1186/1750-1172-10-S1-P52

**Published:** 2015-11-02

**Authors:** Massimo Russo, Gianluca Vita, Claudia Stancanelli, Giuseppe Vita, Anna Mazzeo, Sonia Messina

**Affiliations:** 1 Nemo Clinical Center Messina, University of Messina, 98125, Messina, Italy; 2 Biomedical Department of Internal and Specialistic Medicine, University of Palermo, 90100, Palermo, Italy; 3 Department of Neuroscience, University of Messina, 98125, Messina, Italy

## Background

Transthyretin related familial amyloidotic polyneuropathy (TTR-FAP) is an inherited form of amyloidosis, leading to death in about 10 years in most cases for cardiac failure or wasting syndrome. Previous study showed that modified body mass index (mBMI) was related to time before death, duration of gastrointestinal disturbances, malabsorption and functional capacity. Futhermore, outcome after liver tranplantation was greater in patients with an mBMI over 600.

## Patients

We report two TTR-FAP patients, carrying respectively the Thr49Ala and the Glu89Gln mutations, in whom nutritional status worsened despite diet modification, hypercaloric supplement and two important therapeutic approaches such as liver transplant and tafamidis meglumine.

In both case, at a late stage of the disease, a peripherally inserted central catheter (PICC) was placed and the parenteral nutrition started.

## Results

The parenteral nutrition added to oral nutrition allowed to improve their nutritional status and clinical conditions as documented by increase of body weight and mBMI. Moreover, we recorded reduction of autonomic symptoms including postural hypotension, nausea and diarrhoea and amelioration of quality of life.

## Conclusion

Our experience suggests that parenteral nutrition administered by PICC may be useful in reducing complications and disabilities in TTR-FAP patients, even when all dietary adjustments have been ineffective. Reasonably, the improvement in nutritional status may prolong survival in TTR-FAP patients.

